# Estimating Flight Characteristics of Anomalous Unidentified Aerial Vehicles

**DOI:** 10.3390/e21100939

**Published:** 2019-09-25

**Authors:** Kevin H. Knuth, Robert M. Powell, Peter A. Reali

**Affiliations:** 1 Department of Physics, University at Albany (SUNY), Albany, NY 12222, USA; 2 Scientific Coalition for UAP Studies (SCU), Fort Myers, FL 33913, USA; robertmaxpowell@gmail.com (R.M.P.); preali@cableone.net (P.A.R.)

**Keywords:** UAP, UAV, UFO, Nimitz, Tic-Tac

## Abstract

Several Unidentified Aerial Phenomena (UAP) encountered by military, commercial, and civilian aircraft have been reported to be structured craft that exhibit ‘impossible’ flight characteristics. We consider a handful of well-documented encounters, including the 2004 encounters with the *Nimitz* Carrier Group off the coast of California, and estimate lower bounds on the accelerations exhibited by the craft during the observed maneuvers. Estimated accelerations range from almost 100g to 1000s of gs with no observed air disturbance, no sonic booms, and no evidence of excessive heat commensurate with even the minimal estimated energies. In accordance with observations, the estimated parameters describing the behavior of these craft are both anomalous and surprising. The extreme estimated flight characteristics reveal that these observations are either fabricated or seriously in error, or that these craft exhibit technology far more advanced than any known craft on Earth. In many cases, the number and quality of witnesses, the variety of roles they played in the encounters, and the equipment used to track and record the craft favor the latter hypothesis that these are indeed technologically advanced craft. The observed flight characteristics of these craft are consistent with the flight characteristics required for interstellar travel, i.e., if these observed accelerations were sustainable in space, then these craft could easily reach relativistic speeds within a matter of minutes to hours and cover interstellar distances in a matter of days to weeks, proper time.

## 1. Introduction

Unidentified Aerial Phenomena (UAPs) partially identified as being unknown anomalous aircraft, referred to as Unidentified Anomalous Vehicles (UAVs) or Unidentified Flying Objects (UFOs), have been observed globally for some time [1]. Such phenomena were studied officially by the United States Air Force in a series of projects: Project Sign (1947), Project Grudge (1949) and Project Blue Book (1952–1969) [2]. Other nations, such as Australia, Brazil, Canada, Chile [3], Denmark, France, New Zealand, Russia (the former Soviet Union), Spain, Sweden, the United Kingdom, Uruguay, and the Vatican have also conducted studies, or are currently studying, UAPs [4]. In December of 2017 it was revealed that the United States government had been studying UAPs through at least one secret program called the Anomalous Aerospace Threat Identification Program (AATIP) [5], and that there have been times at which United States Naval pilots have had to deal with nearly daily encounters with UAVs [6,7]. These unidentified craft typically exhibit anomalous flight characteristics, such as traveling at extremely high speeds, changing direction or accelerating at extremely high rates, and hovering motionless for long periods of time. Furthermore, these craft appear to violate the laws of physics in that they do not have flight or control surfaces, any visible means of propulsion apparently violating Newton’s Third Law, and can operate in multiple media, such as space (low Earth orbit), air, and water without apparent hindrance, sonic booms, or heat dumps [4].

The nature, origin, and purpose of these UAVs are unknown. It is also not known if they are piloted, controlled remotely, or autonomous. It has been made clear by U.S. officials that if these craft were hostile, then they would pose a serious threat [4]. If some of these UAVs are of extraterrestrial origin, then it would be important to assess the potential threat they pose. More interestingly, these UAVs have the potential to provide new insights into aerospace engineering and other technologies [8]. The potential of a serious threat, the promise of advancements in science and engineering, evolving expectations about extraterrestrial life, and even a deeper understanding of the acts of misperception and misinterpretation are all important reasons for scientists to seriously study and understand these objects [9,10,11,12,13].

In this paper, we carefully examine several well-documented encounters with UAVs, and estimate lower bounds on their accelerations. We demonstrate that the estimated accelerations are indeed extraordinary and surprising. While one cannot prove that any one of these craft is extraterrestrial in origin, we show that their observed accelerations are consistent with accelerations required for effective interstellar travel.

## 2. Case Studies

We consider a handful of case studies of encounters with UAVs. These encounters were selected from a subset of cases for which there were multiple professional witnesses observing the UAV in multiple modalities (including sight, radar, infrared imaging, etc.). This subset was selected based on the fact that there was sufficient information to estimate kinematic quantities such as speeds and accelerations. Due to the professional standing and expertise of the witnesses, and the fact of both qualitative and quantitative agreement among a significant number of witnesses employing different imaging modalities, it is assumed that the relevant details of the events were not fabricated or embellished. Of course, in most situations, one cannot rule out such possibilities. However, it is unlikely that this would occur with multiple independent witnesses. Assuming that any one of the cases we examine is based on accurate reports, we show that the UAVs exhibit unreasonably high accelerations ranging from 100g to well over 5000g.

To properly estimate lower bounds on the observed accelerations of the UAVs, we assign uncertainties to the observations. Unfortunately, such uncertainties are difficult to assign. We assign rather liberal uncertainties modeled by a Gaussian distribution. In some cases, to provide an even more conservative estimate, we integrate (marginalize) over all possible values of σ.

### 2.1. Bethune Encounter (1951)

On 21 February 1951, Lt. Graham Bethune of the U.S. Navy, experienced with 4150 Navy flight hours and 1340 civilian flight hours, was flying Navy R5D, Bureau No. 56501 with Lieutenant Commander (LCDR) Fred Kingdon and Lt. Noel Koger, on a scheduled eight hour passenger flight from Keflavik, Iceland to Argentia, Nova Scotia, while two other crews slept on board. It was a clear northern night, and they were flying on autopilot at 10,000 ft with a ground speed of over 200knots. Lt. Bethune and LCDR Kingdon were on watch for other aircraft. About four and a half hours out of Keflavik, Lt. Bethune noticed a yellow glow below the horizon approximately 30 to 35 miles away that appeared to be city lights. Concerned that they were off course, they had Lt. Koger confirm the navigation and verify that there were no ships in the area. Lt. Jones and Lt. Meyer were woken and came forward into the cockpit. The consensus was that the lights were probably due to a ship. When the lights were about 5 to 7 miles away about $30^{\circ }$ to the right, the lights went out and a circular yellow halo appeared on the water. The halo changed from yellow to orange and then to a fiery red when it rose suddenly to meet them, turning to a blueish red around the perimeter. It arrived at about 100 to 200 feet below their altitude in a fraction of a second [14] and about 200 to 300 feet in front of the airplane. The UAV was observed to be a metallic disk-shaped object that was about 200 to 300 feet in diameter. The UAV flew with the airplane for about 5 min, and was witnessed by most of the passengers on board, before leaving at a speed in excess of 1500mph, which was later confirmed to be about 1800mph by Gander Center Radar, Newfoundland, Canada [15]. It should be noted that the airspeed record of 698.505mph was made almost two years later in November of 1952 by General J. Slade Nash flying a North American F-86D Sabre ([16], p. 24).

In Lt. Bethune’s letter to Stuart Nixon (NICAP), he stated that the UAV was about 5 to 7 miles away [15] when it began its ascent, whereas in his interview with Sirius Disclosure, he states that it was about 15 miles away [14]. We were aware only of the 15-mile distance during our oral presentation. The accelerations have been re-analyzed for this paper using the 5 to 7 mile distance to ensure a more conservative lower bound estimate of the acceleration.

**Figure 1:**
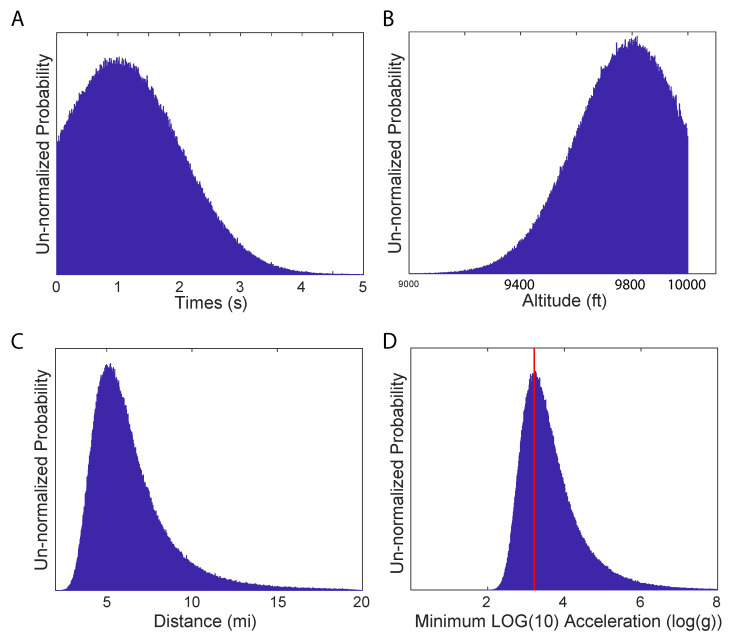
Histograms of the samples used to estimate the minimum acceleration of the UAP in the Bethune encounter. In these and subsequent plots, the y-axis illustrates the number of samples, which is proportional to the probability. (**A**). The duration of the maneuver is a truncated Gaussian distribution for t=1s±1s. (**B**). The altitude of the UAV is a truncated Gaussian with h=9800ft±200ft. (**C**). The horizontal distance traveled was modeled using a Gaussian distribution of angles as described in the text. (**D**). The extreme acceleration calls for a logarithmic scale in the histogram above. The most probable acceleration is approximately $10^{3.23}\approx 1700\;g$.

We employed Monte Carlo sampling to estimate the acceleration of the UAV. The UAV was described as rising from the sea at a distance of about 5 to 7 miles to the approximate position and altitude of the craft in a fraction of a second. We assign uncertainties to these distances and times to accommodate the possibility that the pilot and witnesses could have been in error. The duration of the maneuver was reported to be a fraction of a second. We modeled this as a truncated Gaussian distribution with a mean of 1s and a standard deviation of 1s, which allowed for the possibility that the maneuver could have taken up to two or more seconds (Figure 1A). The altitude to which the UAV rose was modeled as a Gaussian distribution truncated at 10,000 ft with a mean of 9800 ft and a standard deviation of 200 feet (Figure 1B). At an altitude of h=10,000ft, an object at a distance of d=6mi (31,680 ft) on the sea surface would have been at an angle of

(1) $$\theta =\arctan\frac{h}{d}=\arctan\frac{10000\;ft}{31680\;ft}=17.52^{\circ }$$

below the horizontal. We sampled the distance based on a truncated Gaussian-distributed mean angle of $17.52^{\circ }$ with a standard deviation of $5^{\circ }$. This $5^{\circ }$ standard deviation accounts for an error approximately equal to the angular width of one’s fist extended at arm’s length. The resulting distance samples are illustrated in Figure 1C where it can be seen that potential errors in angle lead to an asymmetric distribution.

The Pythagorean theorem was used to obtain a set of sample distances from the initial position of the UAV to Bethune’s airplane. A lower bound on the acceleration is obtained by assuming that the UAV accelerated at a constant rate for one half of the distance and then decelerated at the same rate for the remaining distance. Here we ignore the acceleration of gravity and assume that the aircraft was at rest, which is reasonable considering the extreme speed of the UAV (At a ground speed of about 200knots, the airplane would have traveled 338ft in that 1s). The motion of the UAV was modeled as

(2) $$\frac{1}{2}r=\frac{1}{2}a{\left(\frac{t}{2}\right)}^{2},$$

which is

(3) $$r=a{\left(\frac{t}{2}\right)}^{2}=\frac{1}{4}at^{2}$$

where $r=\sqrt{d^{2}+h^{2}}$ is the total distance traveled, *t* is the duration of the maneuver, and *a* is the acceleration of the UAV. The samples of *r* and *t* are used to obtain samples of the acceleration *a*. The extreme acceleration made it easier to display the distribution using the base-10 logarithm of the acceleration (Figure 1D). The acceleration of the UAV was at least on the order of $10^{3.23}\approx 1700\;g$.

### 2.2. Probability Densities

We wish to remind the reader that densities (here, probability densities) do not transform like functions, and for this reason the means and modes of the probability densities assigned in the calculations in Section 2.1 (as well as Section 2.3 and Section 2.4) do not transform according to (3) despite the fact that (3) was used to compute the acceleration using the samples. Take a simple example in which we have a parameter *x*, for which we have the probability density p(x) and we wish to use the transform y=f(x) to find the probability density p(y)=p(f(x)), given a function *f*. The probability densities are related by

(4) p(y)|dy|=p(x)|dx|,

which has as its solution

(5) $$p\left(y\right)=p\left(x\right){\left|\frac{dy}{dx}\right|}^{-1}=p\left(x\right){\left|\frac{df}{dx}\right|}^{-1},$$

where $\left|\frac{df}{dx}\right|$ is the Jacobian of the transformation [17] (pp. 69–71) [18] (pp. 72–74). It is easily demonstrated that the modes (most probable value) of p(x) do not map to the modes of p(y).

Take an extreme example of a uniform distribution, p(x)=C, and a function $y=f\left(x\right)=x^{-1}$. It is then the case that p(y) has a mode, whereas p(x) does not. It is also the case that given the means of p(x) and p(y),

〈x〉=∫xp(x)dx〈y〉=∫yp(y)dy,

it is true that

(6) y=f(x)doesnotimply〈y〉=f(〈x〉).

For this reason, one obtains inaccurate results by estimating the acceleration using only the mean values (or the most probable values) of *t*, *h*, and *d* in (3) in Section 2.1. Incidentally, this is the reason that the wavelength of the peak energy in the blackbody spectrum does not directly correspond to the frequency of the peak energy in Wien’s law. Taking into account the uncertainties leads to estimates that are more robust given what is known. One way to do this is by generating a large set of samples of *t*, *h*, and *d* and computing a large set of accelerations *a* using (3) and then working with that sampled distribution. Another way to do this is to work directly with the probability densities as in (10) through (14) in Section 2.4.1.

### 2.3. Japan Air Lines Flight 1628 (1986)

**Figure 2:**
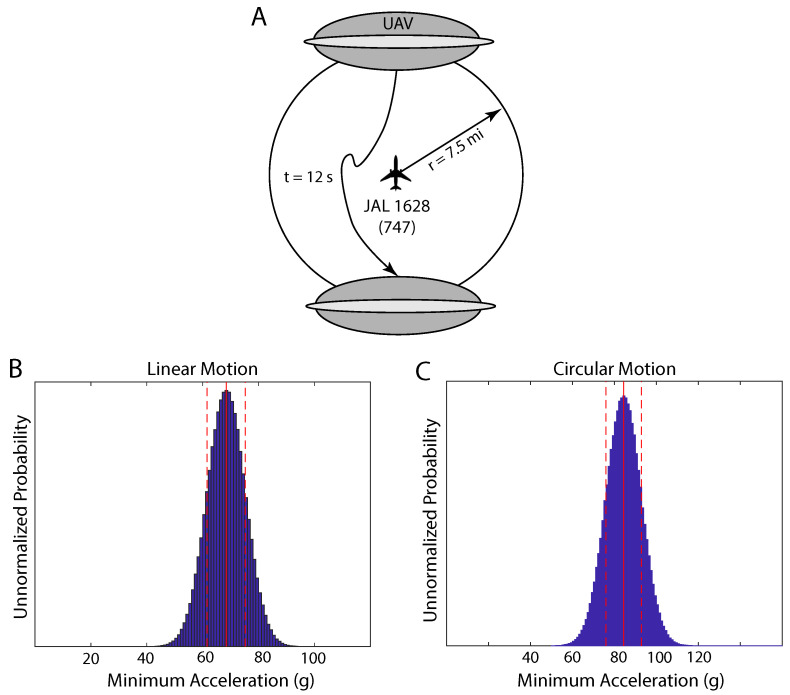
(**A**). An illustration of the behavior of the UAV in the vicinity of JAL 1628. The UAV and the airplane are approximately to scale, while the distance between them is not. (**B**). Modeling the UAV as traveling across the diameter of the circle, the acceleration was estimated to be 68±7g. (**C**). Modeling the UAV as moving in a circular motion and focusing only on the centripetal acceleration, resulted in 84±8g.

On 17 November 1986, Japan Air Lines flight 1628 (JAL 1628) was making its way across Alaska on the Reykjavik to Anchorage leg of a flight from Paris to Tokyo when at 5:00 p.m. north of Anchorage Captain Kenju Terauchi and his crew, Takanori Tamefuji and Yoshio Tsukuda, described seeing two unidentified objects approach their airplane from the left. A larger round UAV, about the size of an aircraft carrier (four Boeing 747s in diameter) with lights running around it, later approached and followed the flight for about 31 min. This large UAV was also tracked on a United States Federal Aviation Administration (FAA) AN/FPS-117 long-range 3-D (azimuth-range-height) phased array antenna radar with a range of 5 to 250 nautical miles and a range precision of less than 50 m [19]. The radar returns revealed that the large UAV stayed about 7.5mi away from the airplane, maintaining that distance as it bounced around the airplane occasionally changing position from one side of the airplane to the other within one 12s radar sweep [20,21] as illustrated in Figure 2A. John Callahan, Division Chief of the Accidents and Investigations Branch of the U.S. Federal Aviation Administration (FAA), has said in several statements that the radar sweep interval was 10s [21], but the radar data itself [20] indicates that the sweep interval was 12s.

We considered two possibilities. In the first case, we considered that the UAV moved linearly across the diameter of the circle accelerating at a constant rate for half of the distance and then decelerating at the same rate for the remaining half (3). The elapsed time was taken to be precisely 12s, which was the sweep rate of the radar. The radius was modeled using Gaussian samples with a mean of 7.50±0.75mi. It should be noted that the assigned uncertainty of 0.75mi, which is 10% of the 7.50mi distance between the airplane and the UAV, is almost four times greater than the FAA Long-Range Radar precision of 0.2mi. As a result, the mean acceleration was estimated to be 68±7g (Figure 2B).

The second possibility was that the UAV traveled around JAL1628 in a circular motion. We considered a situation in which the UAV traveled $180^{\circ }$ and ignored the tangential component of acceleration that would be necessary for the object to accelerate from relative rest at one position and move to another. Focusing only on the centripetal component of acceleration, we have

(7) $$a=\frac{v^{2}}{r},$$

which for a constant velocity

(8) $$v=\frac{\pi r}{t}$$

gives

(9) $$a=\frac{\pi^{2}r}{t^{2}}.$$

This acceleration provides a reasonable lower bound. The radius was modeled using Gaussian samples with a mean of 7.50±0.75mi. The result was a centripetal acceleration of 84±8g (Figure 2C).

### 2.4. Nimitz Encounters (2004)

On 14 November 2004, the U.S. Navy’s Carrier Strike Group Eleven (CSG 11), which includes the USS *Nimitz* nuclear aircraft carrier and the Ticonderoga-class guided missile cruiser USS *Princeton*, was conducting training exercises off the coast of Southern California when the *Princeton*’s radar systems detected as many as 20 anomalous aerial vehicles, which could not be identified. The UAVs were entering the training area and were deemed a safety hazard to the upcoming exercise. The Captain of the USS *Princeton* ordered an interception with two F/A-18F Super Hornet fighter jets. The available data consists of eyewitness information from both the pilots and the radar operators, Freedom of Information Act (FOIA) releases of four Navy documents, and a Defense Intelligence Agency (DIA) released infrared (IR) video of a similar encounter later that day taken by an F/A-18F jet using an AN/ASQ-228 Advanced Targeting Forward Looking Infrared (ATFLIR) system [22]. We estimated the accelerations of the UAVs relying on (1) radar information from USS *Nimitz* former Senior Chief Operations Specialist Kevin Day, (2) eyewitness information from CDR David Fravor, commanding officer of Strike Fighter Squadron 41 and a second jet’s weapons system operator, LCDR Jim Slaight, and (3) analyses of a segment of the DIA-released Advanced Targeting Forward Looking Infrared (ATFLIR) video from an encounter later that day. The following descriptions of the *Nimitz* encounters were summarized from the more detailed study published by the Scientific Coalition for UAP Studies (SCU) [22].

#### 2.4.1. Senior Chief Operations Specialist Kevin Day (RADAR)

An important role of the USS *Princeton* is to act as air defense protection for the strike group. The *Princeton* was equipped with the SPY-1 radar system which provided situational awareness of the surrounding airspace. The main incident occurred on 14 November 2004, but several days earlier, radar operators on the USS *Princeton* were detecting UAPs appearing on radar at about 80,000+ feet altitude to the north of CSG11 in the vicinity of Santa Catalina and San Clemente Islands. Senior Chief Kevin Day informed us that the Ballistic Missile Defense (BMD) radar systems had detected the UAPs in low Earth orbit before they dropped down to 80,000 feet [23]. The objects would arrive in groups of 10 to 20 and subsequently drop down to 28,000 feet with a several hundred foot variation, and track south at a speed of about 100 knots [23]. Periodically, the UAPs would drop from 28,000 feet to sea level (estimated to be 50 feet), or under the surface, in 0.78 s. Without detailed radar data, it is not possible to know the acceleration of the UAPs as a function of time as they descended to the sea surface. However, one can estimate a lower bound on the acceleration, by assuming that the UAPs accelerated at a constant rate halfway and then decelerated at the same rate for the remaining distance as in (2) and (3).

The data consisted of the change in altitude $y\pm \sigma_{y}=8530\pm 90\;m$ (−28,000ft±295ft) and the duration $t^{\prime }\pm \sigma_{t}=0.78\pm 0.08\;s$. The the dominant source of uncertainty in altitude was due to the observed variation in altitude among the observed UAPs, which was on the order of 200 to 300ft leading to our assigned uncertainty of $\sigma_{y}=295\;\mathrm{ft}$. For the duration, we assigned a conservative 10% uncertainty resulting in $\sigma_{t}=0.08\;s$. The goal was to estimate the acceleration, *a*, of the UAP during this maneuver.

In the first analysis, we assigned a joint Gaussian likelihood, P(y,t|a,I) for the measured altitude change, *y*, and the duration, *t*, of the maneuver. Since the altitude change and the duration are independently measured, the joint likelihood is factored into the product of two likelihoods, and one can marginalize over the duration of the maneuver to obtain a likelihood for the altitude *y*

(10) $$\begin{array}{cc} P(y\;|\;a,I) & =\int_{-\infty }^{\infty }dt\;P(y,t\;|\;a,\sigma_{y},t^{\prime },\sigma_{t},I) \end{array}$$

(11) $$\begin{array}{cc} \; & =\int_{-\infty }^{\infty }dt\;P\left(y\;|\;a,t,\sigma_{y},I\right)P\left(t\;|\;t^{\prime },\sigma_{t},I\right), \end{array}$$

where the symbol *I* represents the fact that these probabilities are conditional on all prior information. Assigning Gaussian likelihoods, we have that

(12) $$\begin{array}{cc} P(y\;|\;a,I) & =\int_{-\infty }^{\infty }dt\;\frac{1}{\sqrt{2\pi }\sigma_{y}}\exp\left[-\frac{1}{2\sigma_{y}^{2}}{\left(y+\frac{1}{4}at^{2}\right)}^{2}\right]\frac{1}{\sqrt{2\pi }\sigma_{t}}\exp\left[-\frac{1}{2\sigma_{t}^{2}}{\left(t-t^{\prime }\right)}^{2}\right] \end{array}$$

(13) $$\begin{array}{cc} \; & =\frac{1}{2\pi \sigma_{y}\sigma_{t}}\int_{-\infty }^{\infty }dt\;\exp\left[-\frac{1}{2\sigma_{y}^{2}}{\left(y+\frac{1}{4}at^{2}\right)}^{2}-\frac{1}{2\sigma_{t}^{2}}{\left(t-t^{\prime }\right)}^{2}\right]. \end{array}$$

**Figure 3:**
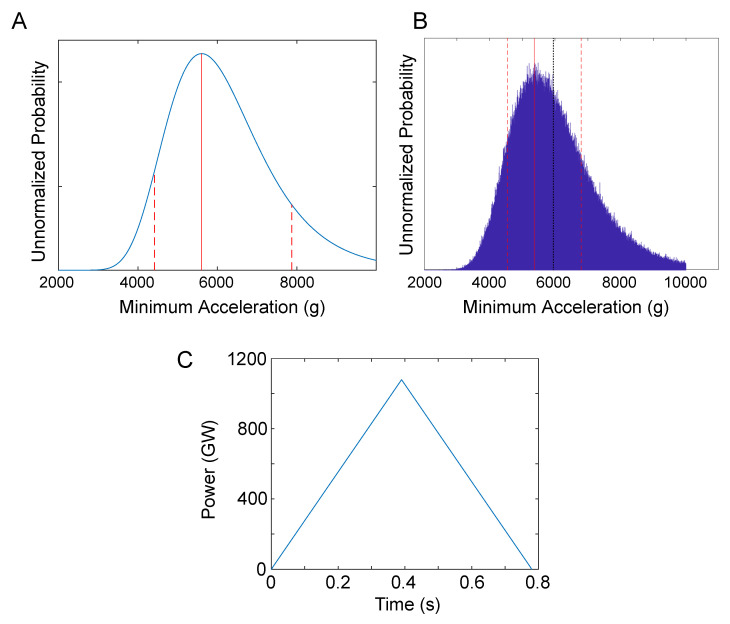
An analysis of Senior Chief Day’s radar observations. (**A**). The posterior probability indicates the maximum likelihood estimate of the acceleration to be $5600_{-1190}^{+2270}$ g. (**B**). The accelerations obtained by sampling resulted in the most probable acceleration of $5370_{-820}^{+1430}$ g while the mean acceleration is 5950g (black dotted line). (**C**). The power output of the UAP, assumed to have a mass of 1000kg, as a function of time indicates a peak power of about 1100GW.

The integrand is the exponential of a quartic polynomial in *t*, which was solved numerically. Assigning a uniform prior probability for the acceleration over a wide range of possible accelerations results in a posterior that is proportional to the likelihood (13) above resulting in a maximum likelihood analysis

(14) $$\begin{array}{cc} P(a\;|\;y,I) & \propto P(y\;|\;a,I)\\ \; & \propto \frac{1}{2\pi \sigma_{y}\sigma_{t}}\int_{-\infty }^{\infty }dt\;\exp\left[-\frac{1}{2\sigma_{y}^{2}}{\left(y+\frac{1}{4}at^{2}\right)}^{2}-\frac{1}{2\sigma_{t}^{2}}{\left(t-t^{\prime }\right)}^{2}\right], \end{array}$$

which gave an estimate of $a=5600_{-1190}^{+2270}$ g, as illustrated in Figure 3A.

We also employed sampling for which the change in altitude and the elapsed time were described by Gaussian distributions with $y\pm \sigma_{y}=8530\pm 90\;m$ and $t^{\prime }\pm \sigma_{t}=0.78\pm 0.08\;s$, respectively. The most probable acceleration was found to be $5370_{-820}^{+1430}$ g while the mean acceleration was found to be 5950g (Figure 3B).

With acceleration estimates in hand, we obtained a ballpark estimate of the power involved to accelerate the UAP. Of course, this required an estimate of the mass of the UAP, which we did not have. The UAP was estimated to be approximately the same size as an F/A-18 Super Hornet, which has a weight of about 32000lbs, corresponding to 14550kg. Since we want a minimal power estimate, we took the acceleration as 5370g and assumed that the UAP had a mass of 1000kg. The UAP would have then reached a maximum speed of about 46000mph during the descent, or 60 times the speed of sound. The power, *P*, required to accelerate the UAP is given by

(15) $$P=Fv=mav=ma^{2}t,$$

for which *F* is the force, *m* is the mass of the UAP, *v* is its velocity, and *a* is its acceleration. The power required varies as a function of velocity, and hence as a function of time. Figure 3C illustrates the power required to accelerate the UAV as a function of time, assuming that the UAV is propelled in a conventional way. The required power peaks at a shocking 1100GW, which exceeds the total nuclear power production of the United States by more than a factor of ten. For comparison, the largest nuclear power plant in the United States, the Palo Verde Nuclear Generating Station in Arizona, provides about 3.3GW of power for about four million people [24].

#### 2.4.2. Commander David Fravor (PILOT)

On 14 November 2004, CSG11 was preparing for training exercises. Two F/A-18F Super Hornets were launched from the *Nimitz* for the air defense exercise to be conducted in an area 80–150 miles SSW of San Diego. Both planes, with call signs “FastEagle01” and “FastEagle02”, had a pilot and a weapons system operator (WSO) onboard. VFA-41 Squadron Commanding Officer David Fravor was piloting FastEagle01 and LCDR Jim Slaight was the WSO for FastEagle02. CDR Fravor and his wingman were headed for the Combat Air Patrol (CAP) point, which is given by predefined latitude, longitude and altitude coordinates, where they would conduct the training exercises.

About a half-hour after take-off, Senior Chief Day operating the SPY-1 radar system on the *Princeton* detected UAVs entering the training area. The training exercise was delayed and FastEagle01 and FastEagle02 were directed to intercept a UAV at a distance of 60 miles and an altitude of 20,000 feet. As the F-18s approached *merge plot*, which is the point at which the radar could not differentiate the positions of the F-18s and the UAV, Fravor and Slaight noticed a disturbed patch of water, where it appeared as if there was a large object, possibly a downed aircraft, submerged 10 to 15 feet below the surface. As they observed the disturbance from 20,000 ft, all four pilots spotted a white UAV, shaped like a large cylindrical butane tank, or a Tic-Tac candy, moving erratically back and forth, almost like a bouncing ping-pong ball making instantaneous changes in direction without changing speed. The Tic-Tac UAV was estimated to be about the size of an F-18, about 56 feet in length and 10–15 feet wide, but had no apparent flight surfaces or means of propulsion, and its movement had no apparent effect on the ocean surface as one would expect from something like rotor wash from a helicopter.

Fravor started a descent to investigate while his wingman kept high cover. As Fravor circled the area and descended, the UAV seemed to take notice of him and rose to meet him. The F-18 and the UAV circled one another. When Fravor reached the nine o’clock position, he performed a maneuver to close the distance by cutting across the circle to the three o’clock position. As he did so, the Tic-Tac UAV accelerated ([22], p. 12) across Fravor’s nose heading south. Fravor said that the UAV was gone within a second. As a comparison, Fravor noted that even a jet at Mach 3 takes 10 to 15 s to disappear from sight ([22], p. 11). LCDR Slaight described the UAV as accelerating as if it was “shot out of a rifle” and that it was out of sight in a split second ([22], p. 12)

The engagement lasted five minutes. With the Tic-Tac gone, the pilots turned their attention toward the large object in the water, but the disturbance has disappeared. The two FastEagles returned to the *Nimitz*, with insufficient fuel to attempt to pursue the Tic-Tac. On their way back, they received a call from the *Princeton* that the Tic-Tac UAV was waiting precisely at their CAP point. Senior Chief Day noted that this was surprising because those coordinates were predetermined and secret. Given that the CAP point was approximately R=60mi away, the probability of selecting the CAP point out of all the locations within the 60 mile radius, to within a one mile resolution (slightly more than the resolution of the radar system), is

(16) $$P\left(x|I\right)=\frac{1}{\pi R^{2}}=\frac{1}{11310}=0.0088\%,$$

discounting the altitude. Given the improbability of this being a coincidence, it appears that the Tic-Tac UAV intentionally went to their CAP point, although it is neither clear how the UAV determined the CAP point coordinates nor why it would perform such a maneuver. However, it should be noted that the UAV was not observed on radar moving to the CAP point, but that it was discovered that a UAV had moved to the CAP point just after the encounter. Since there were several UAVs in the area at the time, it is not clear that it was the specific UAV that CDR Fravor encountered, but it was one of the UAVs in the area.

**Figure 4:**
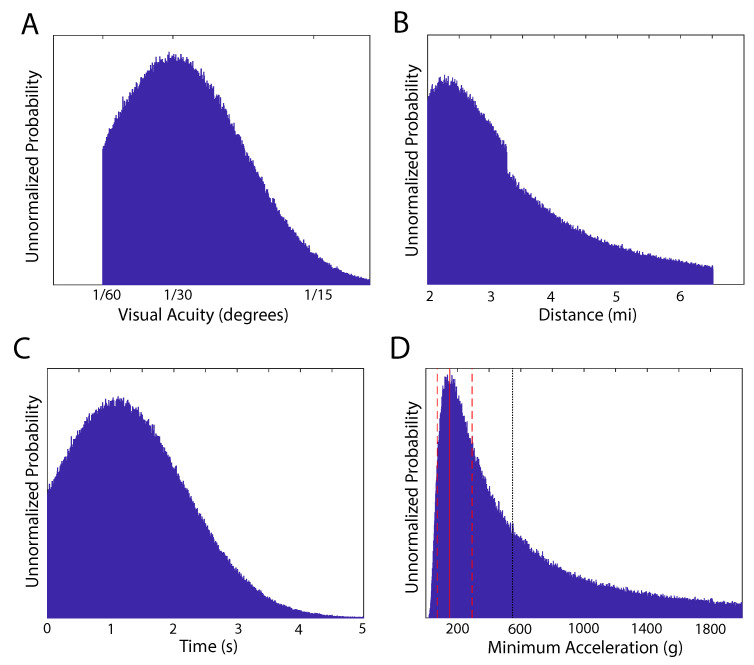
An analysis of CDR Fravor’s encounter. (**A**). Truncated Gaussian distribution of Fravor’s visual acuity based on ${1/30}^{\circ }\pm {1/60}^{\circ }$. (**B**). Gaussian distribution of distances based on the visual acuity distribution in A. (**C**). The distribution of times based on 1±1s. (**D**). The distribution of accelerations has a maximum at $150_{-80}^{+140}$ g (red lines) and a mean of 550g (black dotted line).

To obtain a lower bound on the acceleration, we assume that the UAV exhibited constant acceleration so that the distance *d* traveled is given by

(17) $$d=\frac{1}{2}at^{2}$$

during the elapsed time. The length of the Tic-Tac UAV was estimated to be about 40ft with a cross sectional width of about w=10ft. Given that the acuity of human vision is about $\theta =1/60^{\circ }$ the UAV, at its narrowest, would be out of sight at a maximum distance of

(18) $$d=\frac{w/2}{tan(\theta /2)},$$

which is d≈6.5mi. It is difficult to know what Fravor’s acuity was given the viewing conditions. For this reason, we model the acuity conservatively as a truncated Gaussian distribution with a peak at $\theta =1/30^{\circ }\pm 1/60^{\circ }$ (Figure 4A). The truncation at $\theta =1/60^{\circ }$ resulted in a discontinuity in the distribution of the distances (Figure 4B), which peaks around 2.25mi.

The elapsed time is modeled as a Gaussian distribution with a mean of 1±1s and truncated for positive values of time (Figure 4C). The resulting acceleration distribution was a skewed distribution of accelerations (Figure 4D) with a most probable acceleration of $150_{-80}^{+140}\;g$, indicated in the figure by the red vertical lines and a mean acceleration of about 550g indicated by the black vertical dotted line. Please note that this is a lower bound, probably far below the observed acceleration if the UAV accelerated briefly as if “shot out of a rifle” and then traveled at a constant speed.

#### 2.4.3. ATFLIR Video

**Figure 5:**
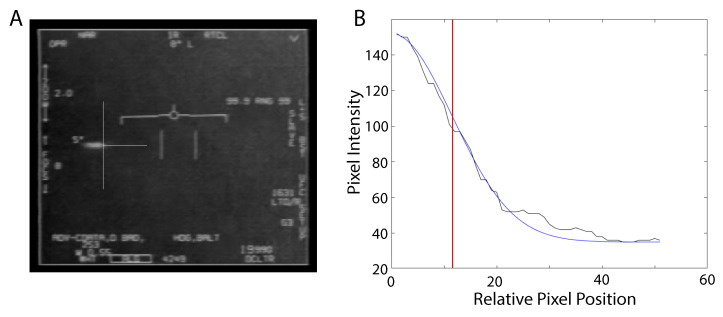
(**A**). Frame 19 of the last 32 frames of the *Nimitz* ATFLIR video. The narrow horizontal and vertical lines intersecting at the right edge of the UAP image indicate the position of the UAP. (**B**). The pixel intensities along a row of the frame are plotted along with the best Gaussian curve fit. The rightmost edge of the craft is defined as the center position of the Gaussian plus one standard deviation (indicated by the vertical red line).

Upon returning to the *Nimitz*, CDR Fravor requested that a crew equipped with the ATFLIR pod obtain videos of the Tic-Tac UAV. Two F/A-18Fs were launched under the guidance of an E-2 Hawkeye airborne radar plane. The two planes separated in search of the UAV, with one plane heading south toward the CAP point where the UAV was last seen on radar. That plane picked up a contact 33 miles to the south on the Range-While-Search (RWS) scan. This Tic-Tac UAV was filmed using the ATFLIR system, and the video was released to the public as the “*Nimitz* video” (Figure 5A) [25].

We examined the last 32 frames of the *Nimitz* video in which the Tic-Tac UAV accelerated to the left and the targeting system lost lock. The video frame rate was 29.97frames/s. From 0.267s (8 frames) before the analyzed segment of video through the end of the analyzed segment of video, the aircraft orientation was fixed and the ATFLIR orientation was fixed at a zenith angle of $5^{\circ }$ above the aircraft axis and at an azimuthal angle of $8^{\circ }$ left of the aircraft axis, so that the apparent motion of the UAV in the video frames is attributable only to the physical motion of the UAV. This means that, for the sake of this analysis, the UAV can be treated as if starting from rest with respect to the aircraft.

As the UAV accelerates, the image of the UAV becomes elongated and blurred. If the shutter speed was known, then this information could be used to better estimate the speed of the craft. This could be accomplished by treating the shutter speed as a model parameter, but such analysis is beyond the scope of this project. Instead, we concentrated on tracking the position of the right edge of the UAV and using those positions to estimate the kinematics. The left edge of the UAV was also estimated in the first frame to provide some information about the range, $z_{o}$, to the UAV given that the UAV was estimated to be at least 40 feet in length. However, since the orientation was not known, this is modeled as a uniformly distributed unknown parameter ϕ, which allowed one to at least put an upper bound on the range $z_{o}$. For clarity, it should be noted that the Tic-Tac UAVs were described as being about 40 to 50ft in length or the size of the F-18, which is 56ft. Here we assume that the UAV is 40ft in length, which is probably an underestimate. As such, the estimated accelerations are expected to be underestimated, and thus more conservative.

To estimate the position of the right edge of the craft in each frame (Figure 5A), the row of pixels for which the UAV has a maximum intensity was examined. The pixel intensities along that row at the right edge of the UAP were fit (maximum likelihood method with a Student-t likelihood) to half of a Gaussian curve. The center position of the Gaussian plus the standard deviation was used as the position of the right side of the UAV for that frame (Figure 5B).

**Figure 6:**
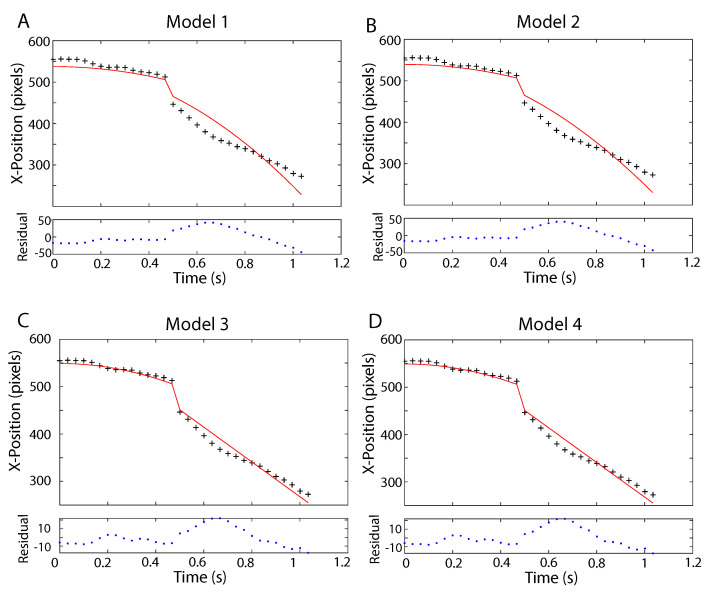
The figures (**A**–**D**) illustrate the position of the right edge of the UAV (+) in pixels, the model fits (solid curves) to the UAV positions in the *Nimitz* ATFLIR video, and the residuals (model minus data) for each of the four models described in (20), (21), (22), and (23), respectively. The model parameter values for each of the models are listed in Table 1 along with the log evidence, logZ, and log likelihood, logL. The log evidence, logZ (Table 1), strongly favors Model 4 (D), which describes the UAV as accelerating at a magnitude of 75.9±0.2g for about 0.53s to the left and away from the observer. Even though the data are well described by Model 4, it appears from the residuals that the UAV may have accelerated and decelerated erratically multiple times.

Horizontal positions of the UAV are related to the pixel coordinates by noting that the entire field of view (approximately $FOV_{pix}=606\;\mathrm{pixels}$) corresponds to an angular field of view of $0.7^{\circ }$ in the narrow (NAR) mode, which is indicated in the upper left hand corner of the video. At the range, $z_{o}$, of the UAV this results in the proportion

(19) $$X_{scale}=\frac{FOV_{pix}/2}{z_{o}\tan\frac{0.7^{\circ }}{2}}$$

where $X_{scale}$ has units $\frac{pixels}{m}$ when $z_{o}$ is in units of m. A similar relation holds for the vertical position of the UAV, but it was not used in this analysis. The ATFLIR has a zoom feature that can change the field of view. In the *Nimitz* video frames analyzed, the zoom is first set to unity in the NAR mode so that the angular field of view is $0.7^{\circ }$. However, at Frame 16, the zoom changes to two, so that the angular field of view in the NAR mode changes to $0.35^{\circ }$. This appears as a discontinuity in the data (‘+’ symbols) illustrated in Figure 6.

We consider several different kinematic models analyzed using nested sampling, and statistically test them by comparing the log Bayesian evidence. The coordinates were defined so that the x-direction corresponds to motion to the left and right, and the z-direction corresponds to motion toward and away from the camera. We used uniform prior probabilities for the kinematic parameters as well as a Student-t likelihood function, which is robust to outliers, such as those due to camera (airplane) motion. The first kinematic model assumes that the UAV started from relative rest and accelerated with a constant rate of $a_{x}$ to the left. The model then provides the position of the UAV as a function of time, where $t_{i}$ is the time of the $i^{th}$ video frame:

(20) $$\mathrm{Model}\;1\;\begin{array}{c} \left\{\begin{array}{cc} x(t_{i}) & =\frac{1}{2}a_{x}{t_{i}}^{2}+x_{o}\\ z(t_{i}) & =z_{o} \end{array}\right.\;\mathrm{constant}\;\mathrm{acceleration}\;\mathrm{in}\;a_{x}, \end{array}$$

so that there are four model parameters: the UAV’s acceleration $a_{x}$, its initial position $x_{o}$, its range $z_{o}$, and its orientation ϕ in the first frame, which helps to set the scale.

The second kinematic model considers constant acceleration in both the *x* and *z* directions:

(21) $$\mathrm{Model}\;2\;\begin{array}{c} \left\{\begin{array}{cc} x(t_{i}) & =\frac{1}{2}a_{x}{t_{i}}^{2}+x_{o}\\ z(t_{i}) & =\frac{1}{2}a_{z}{t_{i}}^{2}+z_{o} \end{array}\right.\;\mathrm{constant}\;\mathrm{acceleration}\;\mathrm{in}\;a_{x}\;\mathrm{and}\;a_{z}. \end{array}$$

The last two models describe the kinematics as acceleration followed by motion at constant velocity:

(22) $$\mathrm{Model}\;3\;\begin{array}{c} \left\{\begin{array}{cc} x(t_{i}) & =\frac{1}{2}a_{x}{t_{i}}^{2}+x_{o}\;\;\;\;\;\;\;\;\mathrm{for}\;\;t_{i}<t_{16}\\ x(t_{i}) & =\frac{1}{2}a_{x}{t_{15}}^{2}+a_{x}t_{15}\left(t_{i}-t_{15}\right)+x_{o}\;\mathrm{for}\;\;t_{i}\geq t_{16}\\ z(t_{i}) & =z_{o} \end{array}\right.\;\mathrm{limited}\;\mathrm{accel}.\;\mathrm{in}\;a_{x} \end{array}$$

and

(23) $$\mathrm{Model}\;4\;\begin{array}{c} \left\{\begin{array}{cc} x(t_{i}) & =\frac{1}{2}a_{x}{t_{i}}^{2}+x_{o}\;\;\;\;\;\;\;\;\mathrm{for}\;\;t_{i}<t_{16}\\ x(t_{i}) & =\frac{1}{2}a_{x}{t_{15}}^{2}+a_{x}t_{15}\left(t_{i}-t_{15}\right)+x_{o}\;\mathrm{for}\;\;t_{i}\geq t_{16}\\ z(t_{i}) & =\frac{1}{2}a_{z}{t_{i}}^{2}+z_{o}\;\;\;\;\;\;\;\;\mathrm{for}\;\;t_{i}<t_{16}\\ z(t_{i}) & =\frac{1}{2}a_{z}{t_{15}}^{2}+a_{z}t_{15}\left(t_{i}-t_{15}\right)+z_{o}\;\mathrm{for}\;\;t_{i}\geq t_{16} \end{array}\right.\;\lim.\;\mathrm{accel}.\;\mathrm{in}\;a_{x}\;\mathrm{and}\;a_{z}, \end{array}$$

in which we consider acceleration in both the *x* and *z* directions until Frame 16, at which time the UAV continues with constant velocity.

The models were analyzed using a nested sampling algorithm [17,26,27], which allowed for the estimation of the logarithm of the Bayesian evidence, logZ, as well as the logarithm of the likelihood, logL, and mean estimates of the model parameters. The analysis was performed for N=500 samples and was run until the change in logZ from successive iterations was less than $10^{-5}$, ensuring a reliable estimate of the log evidence. Tests were performed to ensure that the trial-to-trial variations in parameter estimates were within the estimated uncertainties.

The results of the nested sampling analysis are listed in Table 1. The uncertainties in the logZ estimates (not listed) were on the order of one or less. We see that Model 4, which describes the motion of the UAV as a constant acceleration to the left and away from the observer for the first 15 frames (approximately 0.53s) is the most probable solution with acceleration components of $a_{x}=-35.64\pm 0.08\;g$ and $a_{z}=67.04\pm 0.18\;g$ for an overall acceleration of about 75.9±0.2g. While Model 4 describes the data well, the residuals indicate that a more precise model would consist of multiple episodes of acceleration and deceleration during the maneuver. This was observed in SCU’s analysis [22] where the accelerations were estimated to vary from around 40 to 80 g.

A more detailed analysis would involve modeling the motion of the UAV more precisely by modeling the pixel intensities on the video frames themselves. One could consider the shutter speed of the camera, which would take advantage of the blurring of the UAV image due to its motion while the shutter was open. In addition, the “change points” at which the accelerations changed could be treated as model parameters. This would allow for more precise estimates of the UAV’s behavior.

## 3. Discussion

We have carefully considered a handful of encounters with UAVs of unknown nature and origin. Reports of the encounters have described these UAVs as having “amazing” or “impossible” flight characteristics. In this paper, we objectively quantified the observed accelerations. In some situations, the information available consisted of eyewitness descriptions. However, in each of these cases the eyewitnesses were trained observers, and these encounters were selected because they involved multiple witnesses observing in multiple modalities including visual contact from pilots and passengers, radar, and infrared video. While fabrication and exaggeration cannot be ruled out, the fact that multiple professional trained observers working in different modalities corroborate the reports greatly minimizes such risks.

The facts that the estimated accelerations of encounters spanning over 50 years all fall within two orders of magnitude of one another and that they are far greater in magnitude than one would expect serve to further minimize the risks of fabrication or exaggeration. Furthermore, our acceleration estimates are similar to previous estimates of accelerations measured in other encounters, such as the accelerations ranging from $175\;m/s^{2}$ to $4407\;m/s^{2}$ (17.9g to 450g) estimated from radar data obtained during the 1968 Minot AFB encounter in North Dakota, USA [28]. In addition, the German physicist Hermann Oberth, one of the founding fathers of astronautics and rocketry, gave a lecture on UFOs in 1954 in which he reported the top measured speed to be 19km/s [29], or Mach 55, which is comparable to the maximum speed of ∼Mach 60 we estimated in Section 2.4.1 from the radar observations of Senior Chief Day on the USS *Princeton* during the 2004 *Nimitz* encounters.

The analyses we performed aimed to estimate lower bounds on the acceleration. This was found by assuming that the UAVs accelerated a constant rate. We worked to obtain conservative estimates by assigning liberal uncertainties. It was found that the minimum acceleration estimates far exceeded (often by orders of magnitude) those expected for an aircraft. A summary of the estimated accelerations is provided in Table 2. The observed UAV accelerations range from about 70g to well over 5000g. For comparison, humans can endure up to 45g for 0.044s with no injurious or debilitating effects, but this limit decreases with increasing duration of exposure [30]. For durations more than 0.2s the limit of tolerance decreases to 25g and it decreases further still for longer durations [30].

These considerations suggest that these UAVs may not have been piloted, but instead may have been remote controlled or autonomous. However, it should be noted that even equipment can only handle so much acceleration. For example, the Lockheed Martin F-35 Lightning II has maintained structural integrity up to 13.5g [31]. Missiles can handle much higher accelerations. The Crotale NG VT1 missile has an airframe capable of withstanding 50g and can maintain maneuverability up to 35g [32]. However, these accelerations are still only about half of the lowest accelerations that we have estimated for these UAVs. The fact that these UAVs display no flight surfaces or apparent propulsion mechanisms, and do not produce sonic booms or excessive heat that would be released given the hundreds of GigaWatts of power that we expect should be involved (Figure 3C), strongly suggests that these anomalous craft are taking advantage of technology, engineering, or physics that we are unfamiliar with. For example, the Tic-Tac UAV dropping from 28,000 ft to sea level in 0.78s involved at least $4.3\times 10^{11}\;J$ of energy (assuming a mass of 1000kg), which is equivalent to about 100 tons of TNT, or the yield of 200 Tomahawk cruise missiles, released in $\frac{3}{4}$ of a second. One would have expected a catastrophic effect on the surrounding environment. This does not rule out the possibility that these UAVs have been developed by governments, organizations, or individuals on Earth, but it suggests that these UAVs and the technologies they employ may be of extraterrestrial origin.

That being said, it should be strongly emphasized that proving that something is extraterrestrial is extremely difficult, even if one had a craft in hand. One might imagine that the presence of unidentifiable, or incomprehensible, technology would constitute potential evidence. However, it would not rule out the fact that it could have been created by someone on Earth. The purpose of this paper is not to prove the Extraterrestrial Hypothesis, but instead to focus on the flight kinematics of these UAVs with the aim of building up a body of scientific evidence that will allow for a more precise understanding of their nature and origin.

**Figure 7:**
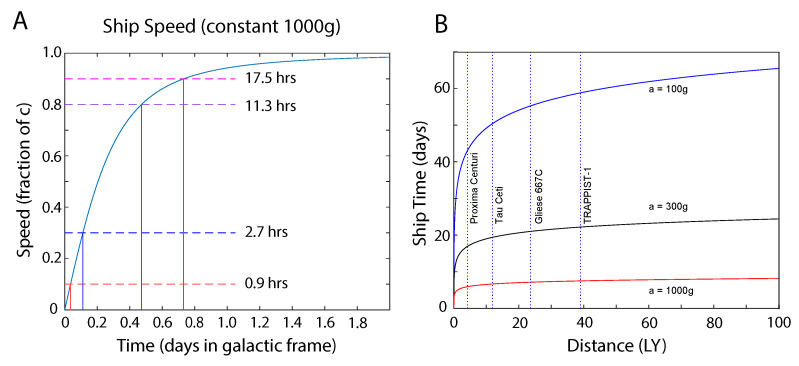
(**A**). This figure shows the time required to reach relativistic speeds for a craft undergoing constant acceleration at 1000g. In less than 24hrs, such a craft would exceed 90% the speed of light. (**B**). This figure shows the travel time to various distances assuming that the craft accelerates at a constant rate for half of the trip and decelerates at the same rate for the second half. The four star systems indicated are each believed to host one or more planets within the habitable zone. At an acceleration of 100g a craft could travel to Proxima Centuri, 4.37LY distant, in about one and a half months for the travelers. For those of us on Earth, or anywhere else in the galactic frame, the trip would take over four years.

While the Extraterrestrial Hypothesis can be neither verified nor ruled out at this time, it is useful to consider whether the characteristics of these UAVs tend to support or rule out the Extraterrestrial Hypothesis. Given the estimated accelerations of these UAVs, it is useful to consider the time it would take them to travel interstellar distances. Figure 7A illustrates how long it would take a craft accelerating at 1000g to reach various percentages of the speed of light. In just less than an hour, a craft accelerating at a constant 1000g would reach 10% of the speed of light, which is NASA’s goal for the planned 2069 mission to Proxima Centuri [33] (Alpha Centuri system). In less than three hours, the same craft would reach 30% of the speed of light. Such a craft accelerating at a constant 1000g for half of the trip and decelerating at the same rate for the remaining half would reach Proxima Centuri within 5 days’ ship time due to the fact that it would have been traveling at relativistic speeds for most of the trip (Figure 7B). However, for those of us on Earth, or anyone on Proxima Centuri b, the trip would take over four years. As a comparison, a craft accelerating at 100g would reach 10% of the speed of light in 8.5hrs, 30% of the speed of light in just more than a day, and Proxima Centuri in a month and a half.

Table 3 lists the four star systems illustrated in Figure 7B along with their distances from Earth, and travel times assuming that the spacecraft accelerates at the given acceleration for half of the distance and decelerates at the same rate for the remaining half of the distance. These times are computed using the relativistic rocket equations modified so that the traveler accelerates for half of the trip and decelerates at the same rate for the remaining half or the trip. This involves the travel time τ experienced by the travelers given by

(24) $$\tau =2\frac{c}{a}\mathrm{acosh}\left(\frac{ad}{2c^{2}}+1\right),$$

where *a* is the magnitude of the acceleration, *c* is the speed of light, *d* is the distance traveled in the galactic frame, and the two instances of the number 2 account for the fact that the spacecraft accelerates for half of the distance and decelerates for the remaining half. However, those at home on Earth, or anywhere else in the galactic (rest) frame, will see the trip as taking a time *t* given by

(25) $$t=2\sqrt{{\left(\frac{d}{2c}\right)}^{2}+\frac{d}{a}},$$

which is always slightly longer than it would be if one were traveling at the speed of light. At constant acceleration, the speed is given by

(26) $$v=c\tanh\left(\frac{a\tau }{c}\right)=\frac{at}{\sqrt{{\left(\frac{at}{c}\right)}^{2}+1}},$$

which for the accelerations we are considering, very rapidly approaches the speed of light to within a small fraction of a percent.

The main point is that not only are the observed accelerations of these UAVs consistent with those required for interstellar travel, but that some of these UAVs exhibit capabilities suggesting that they could be spacecraft with impressive interstellar capabilities.

## 4. Conclusions

It is difficult to draw any definitive conclusions at this point regarding the nature and origin of these UAVs other than the fact that we have shown that these objects cannot be of any known aircraft or missiles using current technology. We have characterized the accelerations of several UAVs and have demonstrated that if they are craft then they are indeed anomalous, displaying technical capabilities far exceeding those of our fastest aircraft and spacecraft. It is not clear that these objects are extraterrestrial in origin, but it is extremely difficult to imagine that anyone on Earth with such technology would not put it to use. Even though older sightings are less reliable, observations of seemingly similar UAPs go back to well before the era of flight [1]. Collectively, these observations strongly suggest that these UAVs should be carefully studied by scientists [9,10,11,12,13].

Unfortunately, the attitude that the study of UAVs (UFOs) is “unscientific” pervades the scientific community, including SETI (Search for Extraterrestrial Intelligence) [34], which is surprising, especially since efforts are underway to search for extraterrestrial artifacts in the solar system [35,36,37,38,39], particularly, on the Moon, Mars, asteroids [40], and at Earth-associated Lagrange points. Ironically, such attitudes inhibit scientific study, perpetuating a state of ignorance about these phenomena that has persisted for well over 70 years, which is now especially detrimental, since answers are presently needed [41,42,43,44,45,46].

## Figures and Tables

**Table 1:** Kinematic Models for *Nimitz* Video (Model 4 (**bold**) was found to be most probable by a factor of exp(1200) based on the log evidence (logZ) with an overall acceleration of 75.9±0.2g).

Model	logZ	LogL	$a_{x}$ (g)	$a_{z}$ (g)	$x_{o}$ (m)	$z_{o}$ (m)
Model 1	−253,640	−253,614	−71.1±0.7	–	−15.40±0.04	119,700 ± 1200
Model 2	−236,950	−236,287	7.564±0.002	99.994±0.005	−13.36±0.04	12,193 ± 1
Model 3	−53,282	−53,261	−40.2±3.8	–	−4.02±0.05	49,700 ± 4800
**Model 4**	**−52,084**	**−52,031**	**−35.64 ± 0.08**	**67.04 ± 0.18**	**−3.89 ± 0.05**	**43,870 ± 110**

**Table 2:** Summary of Considered Cases (Detection Modalities include: Visual Contact from Multiple Pilots (Vps), Passenger/s Visual Contact (Vpa/s), Radar (R), Infrared Video (IR). Estimated accelerations range from about 68g to well over 5000g).

Case	Year	Detection Modalities	Refs.	Kinematic Model	Figure	Min. Acceleration
Bethune	1951	Vps,Vpas,R	[14,15]	(3)	Figure 1D	1700g
JAL1628	1986	Vps,R	[21]	(3)	Figure 2	68±7g
				(9)	Figure 2	84±8g
*Nimitz*	2004					
	Day	Vps,R	[22]	(3)	Figure 3B	$5370_{-820}^{+1430}$ g
	Fravor	Vps,R	[22]	(17)	Figure 4C	$150_{-80}^{+140}$ g
	ATFLIR	Vps,R,IR	[22]	(23)	Figure 6D	75.9±0.2g

**Table 3:** Distances and Travel Times to Various Star Systems. (For each system, the left column lists the travel time τ (24) experienced by the travelers in units of days (d) and the right column lists the travel time *t* (25) experienced by those in the galactic (rest) frame in units of years (y).)

Acceleration	Proxima Centauri		Tau Ceti		Gliese 667C		TRAPPIST-1	
	4.37 LY		11.9 LY		25.05 LY		39.17 LY	
	τ	t	τ	t	τ	t	τ	t
100g	43.3d	4.389y	50.4d	11.919y	55.3d	23.619y	58.8d	39.019y
300g	17.0d	4.377y	19.4d	11.907y	21.0d	23.607y	22.2d	39.007y
500g	10.9d	4.374y	12.4d	11.904y	13.3d	23.604y	14.0d	39.004y
1000g	6.0d	4.372y	6.7d	11.902y	7.2d	23.602y	7.5d	39.002y
5000g	1.4d	4.370y	1.56d	11.900y	1.66d	23.600y	1.73d	39.000y

## References

[1] Vallee J., Aubeck C. (2010). Wonders in the Sky: Unexplained Aerial Objects from Antiquity to Modern Times.

[2] Unidentified Flying Objects and Air Force Project Blue Book. https://web.archive.org/web/20030624053806/http://www.af.mil/factsheets/factsheet.asp?fsID=188.

[3] CEFAA Comité de Estudios de Fenómenos Aéreos Anómalos. http://www.cefaa.gob.cl/.

[4] Elizondo L. The imminent change of an old paradigm: The U.S. government’s involvement in UAPs, AATIP, and TTSA. Proceedings of the Anomalous Aerospace Phenomena Conference (AAPC 2019) Presentation.

[5] Cooper H., Blumenthal R., Kean L. Glowing Auras and “Black Money”: The Pentagon’s Mysterious U.F.O. Program. https://creativehammer.com/wp-content/uploads/2017/12/171216-disclosure-lite-nyt.pdf.

[6] Stieb M. Navy Pilots Were Seeing UFOs on an Almost Daily Basis in 2014 and 2015: Report. http://nymag.com/intelligencer/2019/05/navy-pilots-are-seeing-ufos-on-an-almost-daily-basis-report.html.

[7] Rogoway T. Recent UFO Encounters with Navy Pilots Occurred Constantly across Multiple Squadrons. https://www.thedrive.com/the-war-zone/28627/recent-ufo-encounters-with-navy-pilots-occurred-constantly-across-multiple-squadrons.

[8] Monzon I. (2019). Tech CEOs Want to Capture UFOs and Reverse Engineer Them. International Business Times.

[9] Hynek J.A. (1972). The UFO Experience: A Scientific Inquiry.

[10] Hill P.R. (1995). Unconventional Flying Objects: A Scientific Analysis.

[11] Sturrock P.A. (1999). The UFO Enigma: A New Review of the Physical Evidence.

[12] Knuth K.H. (2018). Are We Alone? The Question Is Worthy of Serious Scientific Study. The Conversation.

[13] Colombano S.P. New Assumptions to Guide SETI Research. https://ntrs.nasa.gov/archive/nasa/casi.ntrs.nasa.gov/20180001925.pdf.

[14] Greer S. Encounter Over The Atlantic–Graham Bethune. https://www.youtube.com/watch?v=fU6LOfiUJ6Q.

[15] Bethune G. Bethune Letter to Stuart Nixon. NICAP Report. http://www.nicap.org/docs/bethune_nicapfile_01.pdf.

[16] Allward M. (1978). Modern Combat Aircraft 4-F-86 SABRE.

[17] Sivia D.S., Skilling J. (2006). Data Analysis. A Bayesian Tutorial.

[18] Arfken G.B., Weber H.J., Harris F.E. (2013). Mathematical Methods for Physicists: A Comprehensive Guide.

[19] AN/FPS-117 Long-Range Air Surveillance Radars. https://lockheedmartin.com/content/dam/lockheed-martin/rms/documents/ground-based-air-surveillance-radars/FPS-117-fact-sheet.pdf.

[20] FAA (2010). Alaskan Region, Recorded FAA Radar Data. https://www.theblackvault.com/documentarchive/ufo-case-japanese-airlines-jal1628-november-17-1986/.

[21] Callahan J.J., Kean L. (2011). The FAA investigates a UFO event that “never happened”. UFOs: Generals, Pilots, and Government Officials Go On the Record.

[22] Powell R., Reali P., Thompson T., Beall M., Kimzey D., Cates L., Hoffman R. A Forensic Analysis of Navy Carrier Strike Group Eleven’s Encounter with an Anomalous Aerial Vehicle. https://www.explorescu.org/post/nimitz_strike_group_2004.

[23] Day K. (2019). Personal communication.

[24] Palo Verde Nuclear Generating Station. https://en.wikipedia.org/wiki/Palo_Verde_Nuclear_Generating_Station.

[25] TTSA 2004 USS Nimitz FLIR1 Video. https://thevault.tothestarsacademy.com/2004-nimitz-flir1-video.

[26] Skilling J., Fischer R., Dose V., Preuss R., von Toussaint U. (2004). Nested sampling. Bayesian Inference and Maximum Entropy Methods in Science and Engineering, Garching, Germany 2004.

[27] Skilling J. (2006). Nested sampling for general Bayesian computation. Bayesian Anal..

[28] Poher C. Analysis of Radar And Air-Visual UFO Observations on 24 October 1968 at Minot AFB, North Dakota, USA. https://www.explorescu.org/post/analysis-of-radar-and-air-visual-ufo-observations-on-24-october-1968-at-minot-afb-north-dakota-usa.

[29] Oberth H. (1991). Lecture Notes for Lecture about Flying Saucers 1954. The Australian U.F.O. Bulletin, Sept..

[30] Eiband A.M. Human Tolerance to Rapidly Applied Accelerations: A Summary of the Literature. https://ntrs.nasa.gov/archive/nasa/casi.ntrs.nasa.gov/19980228043.pdf.

[31] Kent J. F-35 Lightning II News. http://www.f-16.net/f-35-news-article4113.html.

[32] Army-Technology.com Crotale NG Short Range Air Defence System. https://www.army-technology.com/projects/crotale/.

[33] Wenz J. Exclusive: NASA Has Begun Plans for a 2069 Interstellar Mission. https://www.newscientist.com/article/mg23631576-000-exclusive-nasa-has-begun-plans-for-a-2069-interstellar-mission/#ixzz5uvdfYrHV.

[34] Wright J. (2019). Searches for technosignatures: The state of the profession. arXiv.

[35] Bracewell R. (1960). Communications from superior galactic communities. Nature.

[36] Bracewell R. (1974). Interstellar probes. Interstellar Communication: Scientific Perspectives.

[37] Freitas R.A. (1983). The search for extraterrestrial artifacts (SETA). J. Br. Interplanet. Soc..

[38] Tough A., Lemarchand G. (2004). Searching for extraterrestrial technologies within our solar system. Symposium-International Astronomical Union.

[39] Haqq-Misra J., Kopparapu R. (2012). On the likelihood of non-terrestrial artifacts in the Solar System. Acta Astronautica.

[40] Kecskes C. (2013). Observation of asteroids for searching extraterrestrial artifacts. Asteroids.

[41] Haines R.F. Aviation Safety in America: A Previously Neglected Factor; National Aviation Reporting Center on Anomalous Phenomena (NARCAP). http://www.noufors.com/Documents/narcap.pdf.

[42] Knapp G., Adams M.L. I-Team: Former Sen. Reid Calls for Congressional Hearings into UFOs. https://www.lasvegasnow.com/news/local-news/i-team-former-sen-reid-calls-for-congressional-hearings-into-ufos/.

[43] History.com Are UFOs a Threat to National Security? This ex-U.S. Official Thinks They Warrant Investigation. https://www.history.com/news/chris-mellon-ufo-investigations.

[44] Bender B.P. Senators Get Classified Briefing on UFO Sightings. https://www.politico.com/story/2019/06/19/warner-classified-briefing-ufos-1544273.

[45] Golgowski N. Congress Briefed on Classified UFO Sightings as Threat to Aviator Safety, Navy Says. https://www.huffpost.com/entry/navy-briefs-congress-ufos_n_5d0baf79e4b06ad4d25cf1be.

[46] Lutz E. Congress Is Taking the UFO Threat Seriously. https://www.vanityfair.com/news/2019/06/congress-is-taking-the-ufo-threat-seriously.

